# The Impact of Obesity on Prostate Cancer and Progression to Castration Resistance—Real-World Data from a Romanian Center

**DOI:** 10.3390/jcm14093146

**Published:** 2025-05-01

**Authors:** Theodor Mustață, Dan Corneliu Jinga, Ioana Lazăr, Sorina Carmen Martin, Anca Elena Sîrbu, Simona Fica

**Affiliations:** 1 Department of Endocrinology, Carol Davila University of Medicine and Pharmacy, 020021 Bucharest, Romania; theodor.mustata@drd.umfcd.ro (T.M.); carmen.martin@umfcd.ro (S.C.M.); anca.sirbu@umfcd.ro (A.E.S.); simona.fica@umfcd.ro (S.F.); 2 Department of Medical Oncology, Neolife Medical Center, 013973 Bucharest, Romania; ioana.lazar@neolife.ro; 3 Department of Endocrinology, Elias University and Emergency Hospital, 011461 Bucharest, Romania

**Keywords:** obesity, BMI, prostate cancer, androgen deprivation therapy

## Abstract

**Background**: The primary endpoint was to assess the nutritional status in patients diagnosed with prostate cancer (PCa) in Romania. The secondary endpoint was to analyze the impact of obesity on mortality and disease recurrence. **Methods**: In this retrospective cohort study, we analyzed the files of 354 patients diagnosed with PCa between August 2001 and May 2022 and referred to the Medical Oncology Department of Neolife Medical Center. A total of 257 patients fulfilled the inclusion criteria and were the subject of this study. **Results**: Excess weight was seen in 190 (73.9%) patients, with 119 (46.3%) being overweight and 71 (27.6%) having obesity. Subgroup analysis showed that at diagnosis, patients with obesity (PwO) were younger (*p* = 0.022), had lower PSA (*p* = 0.016), and had lower 10-year all-cause mortality rates (*p* = 0.04) than patients without obesity. Patients with metastases had lower weight (*p* = 0.001) and BMI (*p* = 0.033), higher PSA (*p* < 0.001), Gleason scores (*p* = 0.002), ISUP grade group (*p* < 0.001), and 10-year all-cause mortality rate (*p* < 0.001) than patients without metastases. Weight (AUROC = 0.637, 95% CI: 0.557–0.717, *p* = 0.001; cut-off = 77.5 kg, Se = 52.5%, Sp = 71.2%) and BMI (AUROC = 0.591, 95% CI: 0.507–0.676, *p* = 0.033; cut-off = 23.5 kg/m^2^, Se = 25.4%, Sp = 91.9%) were independent predictors of the presence of metastases at diagnosis. In the androgen deprivation therapy (ADT) group, PwO had a shorter time to castration resistance than patients without obesity (Log Rank test: χ2 = 4.395, *p* = 0.036). **Conclusions**: Weight and BMI are accessible tools that could be useful in determining the presence of metastatic disease. PwO on ADT may develop castration resistant PCa faster than patients without obesity.

## 1. Introduction

Obesity prevalence has been steadily increasing, regardless of age group, socioeconomic status, or ethnicity, reaching epidemic proportions across the European Region, where 59% of adults are living with overweight or obesity [1]. In 2016, estimates from the World Health Organization (WHO) revealed a prevalence of overweight among Romanian men of 64.3% and of 24.9% for obesity [2]. However, according to the recently published World Obesity Atlas, the prevalence of obesity among Romanian men in 2025 exceeded 40% [3]. On the other hand, prostate cancer (PCa) is the second most frequent malignancy diagnosed in men, with over 1.4 million new cases worldwide in 2020 and over 8 thousand new cases in Romania, respectively [4].

Obesity is one of the three main modifiable causes of cancer related to lifestyle, alongside smoking and alcohol consumption. In fact, it has been classified as a cause of at least 13 types of cancer by the International Agency for Research on Cancer (IARC), with the strongest evidence for cancers of the esophagus, corpus uteri, breast, and kidney. However, the IARC working group concluded that there is limited evidence for an association between fatal prostate cancer and excess body weight, as there is inconsistency in the published data regarding the link between them [5]. The mechanisms that link obesity and cancer include the elevated levels of insulin, which can promote carcinogenesis, either directly through insulin receptors or indirectly by lowering IGF-binding proteins and increasing circulating IGF-1 levels, which in turn act on cancer cell receptors. Additionally, obesity comes with a chronic state of inflammation, which can be attributed to altered adipocytokine homeostasis; excess adipose tissue is associated with lower levels of adiponectin, which has anti-inflammatory properties, and higher leptin levels, which promote inflammation. Moreover, obesity alters steroid hormone levels, and the strongest impact is seen in the postmenopausal woman, where the higher levels of estrogen resulting from the aromatization of androgens in the adipose tissue significantly increase the risk of breast cancer [6].

When it comes to the prostate gland, androgens play a crucial role in its growth and development, through the effects of testosterone and dihydrotestosterone on the androgen receptors, whose expression in prostatic tissue has been observed as early as day 18 in mouse embryonic tissue [7]. Naturally, the dependence on androgens is behind one of the pillars of prostate cancer treatment, alongside surgery and radiotherapy, androgen deprivation therapy (ADT). As early as 1941, Charles Huggins showed the beneficial effect of lowering testosterone levels on metastatic PCa, by orchiectomy or by administration of estrogens [8]. Nowadays, the suppression of androgens for reaching castration levels can be achieved in different ways: bilateral orchiectomy, LH-RH agonists, LH-RH antagonists, steroidal or non-steroidal antiandrogens. Unfortunately, disease progression while on ADT is common, a phenomenon called resistance to castration [9].

When looking at body mass index (BMI), some studies have shown that obesity is associated with a lower risk of localized/low-grade PCa and a higher risk of advanced/high-grade PCa [10,11]. It appears that not only BMI, but the presence of the metabolic syndrome as well, is linked to high-grade PCa and worse pathological outcomes after radical prostatectomy (RP), as shown in a meta-analysis published by Gacci et al. [12]. When it comes to ADT for PCa, the adverse effects on the cardiovascular and metabolic profile are well known [13,14,15,16], but there are limited data regarding the impact of obesity on the response to ADT and progression to castration resistance.

Therefore, the primary endpoint of this retrospective study was to assess the nutritional status in patients diagnosed with PCa and referred to a specialized center in Romania. The secondary endpoint was to analyze the impact of obesity on mortality and disease recurrence.

## 2. Materials and Methods

**Figure 1:**
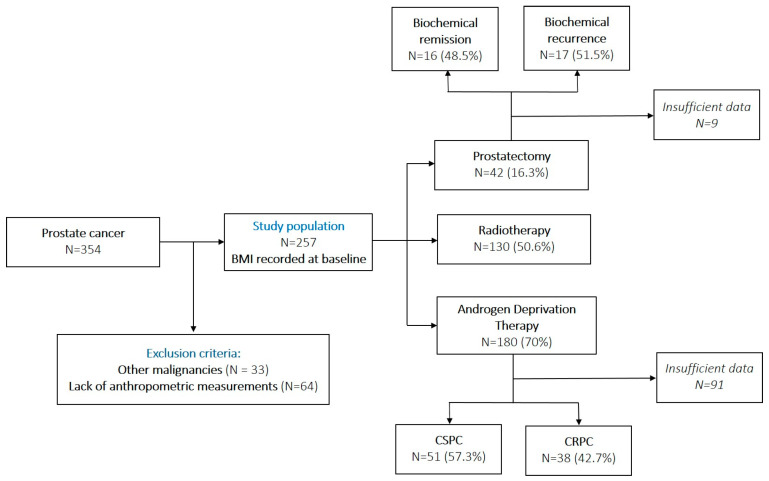
Flow diagram of the patient selection procedure. Insufficient data refer to patients lost to follow-up and/or lack of PSA values.

After obtaining Institutional Review Board approval, we conducted a retrospective cohort study using data from the medical records of 354 patients who were diagnosed with PCa between August 2001 and May 2022 through transrectal prostate biopsy and referred to the Oncology Department of Neolife Medical Center in Bucharest, Romania. Exclusion criteria were prior malignancies (N = 33) and the lack of anthropometric measurements (N = 64), leaving 257 patients to be analyzed. Treatment types included prostatectomy, radiotherapy, and androgen deprivation therapy (Figure 1). They were not mutually exclusive, as some patients received multiple lines of treatment, as follows: 14 patients (5.4%) underwent prostatectomy + radiotherapy; 27 patients underwent prostatectomy + ADT (10.5%); 108 patients received radiotherapy + ADT (42%); 13 patients received all three treatment types (5%). Prior malignancies included colorectal carcinoma (14 cases), bladder carcinoma (11 cases), lung adenocarcinoma (3 cases), non-Hodgkin’s lymphoma (2 cases), breast cancer (1 case), dermatofibrosarcoma (1 case), gastric carcinoma (1 case), pancreatic adenocarcinoma (1 case), renal urothelial carcinoma (1 case), testicular cancer (1 case), thyroid cancer (1 case). It is worth mentioning that there were patients with multiple prior malignancies.

The following clinical and anamnestic data were recorded: age at diagnosis, weight, height, family history for neoplasia, personal history of diabetes, hypertension, and dyslipidemia, respectively, clinical staging according to the American Joint Committee on Cancer’s (AJCC) tumor-node-metastasis (TNM) system [17], and treatment type. Body mass index (BMI) was calculated as weight in kilograms divided by height in meters squared. Using BMI, nutritional status was defined as follows: underweight (BMI < 18.5 kg/m^2^); normal weight (BMI = 18.5–24.9 kg/m^2^); overweight (BMI = 25–29.9 kg/m^2^); obesity (BMI ≥ 30 kg/m^2^). Furthermore, obesity was divided into class I (BMI = 30–34.9 kg/m^2^), class II (BMI = 35–39.9 kg/m^2^), and class III (BMI ≥ 40 kg/m^2^). Weight, circumference, and body composition data were not available. Prostate-specific antigen (PSA) level at diagnosis and at subsequent follow-up visits was noted. Pathological data consisted of cancer type, Gleason Score, and grade group according to the International Society of Urological Pathology (ISUP) [18]. Biochemical recurrence (BCR) for patients who underwent RP was defined as a value of PSA > 0.2 ng/mL at the follow-up visits. All patients who underwent prostatectomy were analyzed for BCR, irrespective of additional treatment lines. Time to BCR was calculated in months as the time from RP to BCR. Patients with proof of disease progression while on androgen deprivation therapy (ADT) were considered as having castration resistant prostate cancer (CRPC). Disease progression was defined as PSA increase, new metastases, or progression of preexisting metastases. For the former, we only looked at patients with at least two follow-up PSA determinations and defined an increase as a PSA doubling time of less than 10 months, or a PSA value ≥ 2 ng/mL above the nadir. Time to castration resistance was calculated in months as the time from ADT start to disease progression. All patients who received ADT and who had follow-up data were analyzed for castration resistance, irrespective of additional treatment lines. Given the indolent course of PCa, we checked the mortality rate at 10 years; because of the retrospective nature of this study and the lack of information regarding the cause of death, 10-year all-cause mortality was assessed.

The Shapiro–Wilk normality test was used to evaluate the distribution of continuous quantitative variables. Parametric variables were presented as means ± standard deviation (SD), while nonparametric variables were presented as medians with interquartile range (IQR). Percentages were used for categorical variables. Comparisons between groups were carried out using an independent sample *t*-test, one-way ANOVA for parametric variables, and the Mann–Whitney U-test, Kruskal–Wallis test for nonparametric variables, respectively. Pearson’s correlation parametric coefficient and Spearman’s rho nonparametric coefficient were used to analyze relations between continuous variables, as appropriate. Binary logistic regression analysis was used to identify the influence of weight and BMI on the risk of death and the likelihood of metastases at diagnosis. Receiver operating characteristic (ROC) curve analysis was used to measure the overall validity of the models with 95% confidence intervals (CI). Kaplan–Meier survival analysis and log-rank test were used for time to recurrence and time to castration resistance, respectively. The statistical analysis was performed using the SPSS statistical package for Windows, version 20.0. (IBM Corp. Released 2011. IBM SPSS Statistics for Windows, Version 20.0. Armonk, NY, USA: IBM Corp.). A *p*-value < 0.05 was used to indicate statistical significance. No multiple testing corrections were applied. The small sample size in some of the analyzed subgroups may widen confidence intervals and lead to *p*-value instability, reducing the statistical power of certain findings.

## 3. Results

Our study population consisted of 257 patients, with a median follow-up period of 24 (38) months (minimum 0, maximum 211). Prostatic adenocarcinoma was the histologic type in 256 cases, while adenocarcinoma with focal neuroendocrine differentiation was found in 1 patient. The clinical and paraclinical characteristics of our study population are described in Table 1.

**Figure 2:**
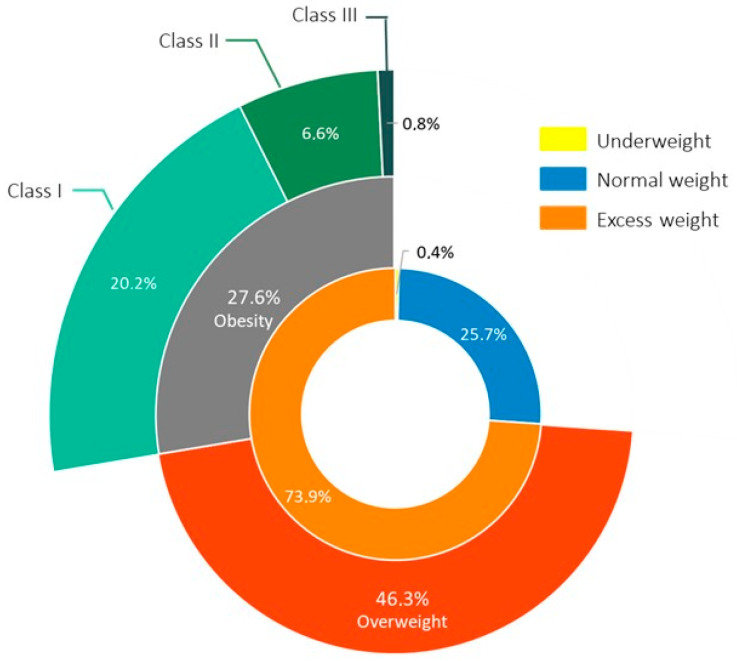
Nutritional status in our study population.

The nutritional status of our subjects is detailed in Figure 2; excess weight was seen in 190 (73.9%) patients, with 119 (46.3%) being overweight and 71 (27.6%) having obesity. Class I obesity was found in 52 (20.2%) patients, class II obesity in 17 (6.6%) patients, and class III obesity in 2 (0.8%) patients. 1 (0.4%) patient was classified as being underweight.

A negative correlation was observed between weight and PSA (ρ = −0.232, *p* < 0.001) and BMI and PSA, respectively (ρ = −0.195, *p* = 0.003). The correlations retained their statistical significance when adjusting for age (weight: ρ = −0.232, *p* < 0.001; BMI: ρ = −0.193, *p* = 0.004). Univariate comparative analysis according to nutritional status (Table 2) showed that patients with obesity (PwO) were diagnosed at younger ages than patients without obesity (65.25 ± 6.96 vs. 67.82 ± 8.34 years, *p* = 0.022) and had significantly lower PSA values at diagnosis (10.13 (22.71) vs. 14.75 (34.73) ng/mL, *p* = 0.016).

**Figure 3:**
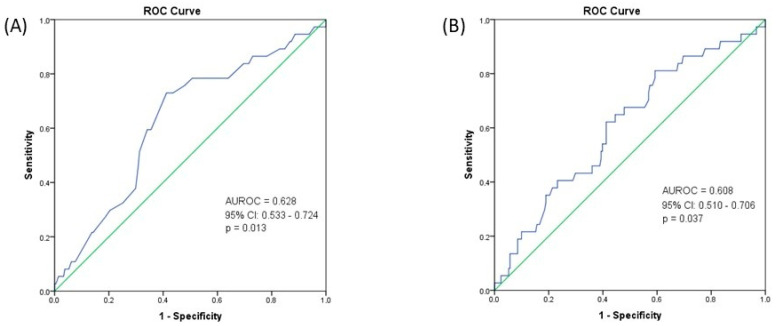
(**A**) Receiver operating characteristic (ROC) curve for the predictive value of weight on 10-year survival. (**B**) ROC curve for the predictive value of BMI on 10-year survival.

Interestingly, there was a lower 10-year all-cause mortality rate in the obesity subgroup (7.4% vs. 17.8%, χ2 = 4.226, *p* = 0.04). Binary logistic regression analysis was performed to assess the effects of obesity on the 10-year survival rate. The logistic regression model was statistically significant, χ2 = 4.762, *p* = 0.029. The model explained 3.3% (Nagelkerke R2) of the variance in the 10-year survival rate and correctly classified 85.1% of cases. PwO had a lower risk of death (OR = 0.367, 95% CI: 0.137–0.985, *p* = 0.047). There was a good capacity of weight (AUROC = 0.628, 95% CI: 0.533–0.724, *p* = 0.013; cut-off = 80.5 kg, Se = 73%, Sp = 58.8%) (Figure 3A) and BMI (AUROC = 0.608, 95% CI: 0.510–0.706, *p* = 0.037; cut-off = 28 kg/m^2^, Se = 81.1%, Sp = 40.8%) (Figure 3B) to predict the 10-year survival.

However, when adjusting for the presence of metastases, the model lost its significance. Univariate comparative analysis according to M stage (Table 3) showed that patients diagnosed in M1 stage had a lower weight (76 (16) vs. 83.5 (21) kg, *p* = 0.001) and BMI (26.3 (5.54) vs. 27.14 (5.98) kg/m^2^, *p* = 0.033) than patients with no metastases. Additionally, the former category had higher PSA values at diagnosis (*p* < 0.001), Gleason scores (*p* = 0.002), ISUP grade group (*p* < 0.001), and 10-year all-cause mortality rate (*p* < 0.001).

**Figure 4:**
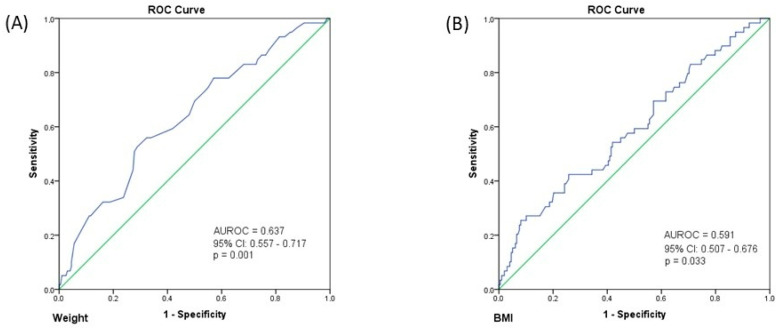
(**A**) ROC curve for the predictive value of weight on the presence of metastases at diagnosis; (**B**) ROC curve for the predictive value of BMI on the presence of metastases at diagnosis.

Binary logistic regression analysis was performed to assess the association between weight and the presence of metastases at diagnosis. The logistic regression model was statistically significant, χ2 = 10.076, *p* = 0.002. The model explained 5.8% (Nagelkerke R2) of the variance in M1 stage presence and correctly classified 77% of cases. An increase in weight was associated with a decreased likelihood of metastases at diagnosis (OR = 0.964, 95% CI: 0.942–0.988, *p* = 0.003). Similarly, binary logistic regression analysis was performed to assess the effects of BMI on the probability of metastases at diagnosis. The logistic regression model was statistically significant, χ2 = 5.758, *p* = 0.016. The model explained 3.4% (Nagelkerke R2) of the variance in M1 stage presence and correctly classified 77% of cases. An increase in BMI was associated with a decreased likelihood of metastases at diagnosis (OR = 0.916, 95% CI: 0.849–0.988, *p* = 0.022). There was a good capacity of weight (AUROC = 0.637, 95% CI: 0.557–0.717, *p* = 0.001; cut-off = 77.5 kg, Se = 52.5%, Sp = 71.2%) (Figure 4A) and BMI (AUROC = 0.591, 95% CI: 0.507–0.676, *p* = 0.033; cut-off = 23.5 kg/m^2^, Se = 25.4%, Sp = 91.9%) (Figure 4B) to predict the presence of metastases at diagnosis.

We divided patients who underwent RP based on the biochemical response. PSA follow-up data were available for 33 patients. BCR was seen in 17 patients. Median time to recurrence was 27.5 (48) months. Univariate comparative analysis according to biochemical response after RP (Table 4) shows no difference between age (*p* = 0.359), mortality (*p* = 0.965), PSA (*p* = 0.081), Gleason score (*p* = 0.526), grade group (*p* = 0.599), weight (*p* = 0.465), BMI (*p* = 0.127) and obesity rate (*p* = 0.161) at diagnosis between patients in remission and patients with BCR after RP. Kaplan–Meier survival analysis showed that obesity had no impact on time to BCR after RP (*p* = 0.202).

**Figure 5:**
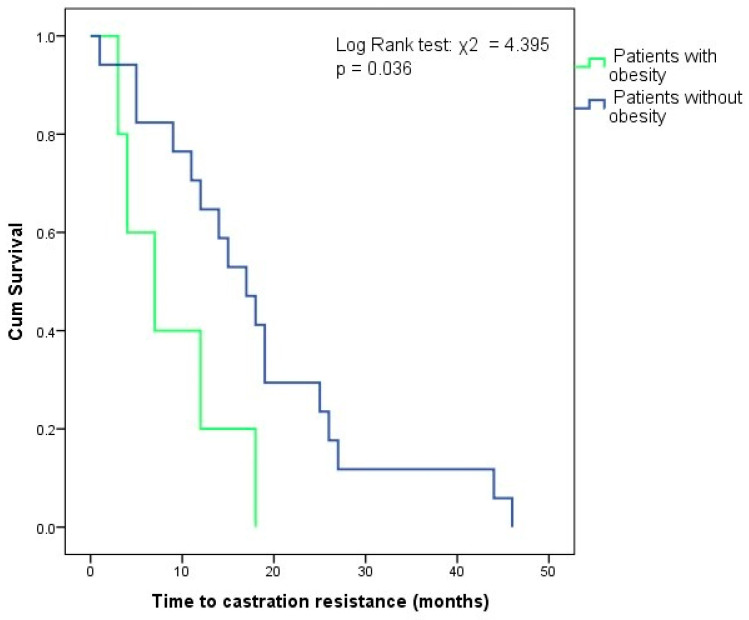
Kaplan–Meier survival analysis of time to castration resistance based on nutritional status.

We divided patients with ADT based on progression on treatment into two groups, castration resistant prostate cancer (CRPC) and castration sensitive prostate cancer (CSPC); 89 patients had the required follow-up data in order to be classified. CRPC was seen in 38 patients. Median time to castration resistance was 14.5 (14) months. Univariate comparative analysis according to response to ADT (Table 5) showed that patients who developed CRPC had higher 10-year all-cause mortality rates (37.1% vs. 4.3%, χ2 = 14.517, *p* < 0.001), higher PSA (63.53 (132.8) vs. 14.9 (26.4) ng/mL, *p* = 0.001), higher Gleason scores (8 (2) vs. 7 (1), *p* = 0.006) and higher prevalence of grade groups IV and V (55.3% vs. 27.5%, χ2 = 10.878, *p* = 0.001) at diagnosis than patients with CSPC. Weight (*p* = 0.243), BMI (*p* = 0.709), and obesity prevalence (*p* = 0.080) did not differ between the two groups. However, Kaplan–Meier survival analysis (Figure 5) showed that in the CRPC group, PwO had a shorter time to castration resistance than patients without obesity (Log Rank test: χ2 = 4.395, *p* = 0.036).

## 4. Discussion

In this retrospective study, we analyzed the prevalence of obesity in 257 patients diagnosed with PCa and the impact it has on mortality and disease recurrence. The prevalence of obesity in our study group was 27.6%. This is in accordance with another retrospective Romanian study on 219 patients with PCa, in which 29% of the subjects were PwO [19]. Similarly, data from the WHO revealed that in 2016, 24.9% of Romanian men were living with obesity [2], while the PREDATORR study had a slightly higher prevalence of obesity, with 29.4% of men falling into this category [20]. A recent analysis from the PIONEER network, which pooled data on 123,146 PCa patients from databases across Europe and the United States, showed a prevalence of obesity in this group ranging from 9.2% to 54% [21].

We found lower PSA levels in PwO, a finding that has been well documented both in cancer patients [22,23] and in healthy subjects [24,25]. Currently, the most accepted hypotheses on this matter are the effect of hemodilution and the lower serum testosterone levels seen in PwO. A higher BMI is associated with a larger blood volume, which dilutes the PSA secreted by the epithelial cells of the prostate. On the other hand, the lower testosterone levels and higher estrogen/testosterone ratio seen in PwO lead to lower PSA levels, owing to the crucial role testosterone and dihydrotestosterone play in the development and growth of the prostate gland [26,27]. Naturally, this raises concerns about the implications for the diagnosis and prognosis of PCa in PwO. Lower PSA levels can delay the referral to biopsy, thus increasing the risk of detecting the disease in an advanced stage. Similarly, disease monitoring may be impaired, potentially delaying the detection of metastases or disease recurrence. Consequently, special considerations could be adopted in PwO, such as adjusting PSA levels for weight/BMI/body surface area, incorporating the clinical findings of digital rectal exam, and the imagistic findings from multiparametric MRI or prostate ultrasound.

Interestingly, we found a lower 10-year all-cause mortality rate among PwO of 7.4% versus 17.8% among patients without obesity. Moreover, using binary logistic regression analysis, we observed a lower risk of death among PwO. Using ROC analysis, we found a cut-off value of 80.5 kg for weight to predict 10-year survival with a sensitivity of 73% and a specificity of 58.8%, and a cut-off value of 28 kg/m^2^ for BMI to predict the 10-year-survival with a sensitivity of 81.1% and a specificity of 40.8%. This finding contradicts the results of a recently published meta-analysis of 280,199 patients, which showed that obesity was associated with increased PCa-specific mortality and all-cause mortality, with a 9% increase in the former and a 3% increase in the latter for each 5 kg/m^2^ increase in BMI [28]. One could bring into discussion the “obesity paradox”, in which a higher BMI may confer a survival advantage in patients with several chronic diseases, including cardiovascular disease [29,30], chronic kidney disease [31], respiratory disease [32,33], diabetes [34], and different types of cancer [35]. When it comes to PCa, the obesity paradox has been brought into discussion in metastatic CRPC, where PwO had better overall survival and cancer-specific survival, irrespective of the chemotherapy doses [36]. However, given the retrospective nature of our study, it is safe to assume that different confounders were associated with this outcome. In this regard, we hypothesized that the presence of metastases at diagnosis is associated with lower weight through disease progression and systemic deterioration. Indeed, when adjusting for the M stage, our model lost its significance, most likely attributing the higher mortality rate in the non-obese group to the presence of metastases. We found that an increase in both BMI and weight was associated with a decreased likelihood of metastases. Using ROC analysis, we found a cut-off value of 77.5 kg for weight to predict the presence of metastases at diagnosis, with a sensitivity of 52.5% and a specificity of 71.2% and a cut-off value of 23.5 kg/m^2^ for BMI to predict the presence of metastases with a sensitivity of 25.4% and a specificity of 91.9%. A comprehensive Swedish case-control study on 1355 PCa patients that looked at anthropometric measurements at multiple time-points in life revealed that an increase in BMI and weight at early middle age (44–50 years old) increased the risk of metastases at 70 years of age. However, a U-shaped relationship was found between BMI and the risk of metastases, with underweight patients exhibiting a higher risk than normal-weight patients [37]. In addition, there is evidence suggesting that obesity is associated with lymph node metastases after RP, owing to the deranged prostatic microenvironment and cellular DNA caused by inflammatory factors (IL-6, IL-8, VEGF, leptin) and altered insulin-IGF-1 axis [38,39,40].

The aforementioned prostatic microenvironment in PwO has also been hypothesized to be a risk factor for BCR after RP, as shown in a recently published meta-analysis on 86,490 PCa patients, where a 5 kg/m^2^ increase in BMI corresponded with a 10% increase in BCR [41]. For this reason, we looked at our patients who underwent RP, and out of the 33 subjects with follow-up data, 17 had BCR, after a median time of 27.5 months. Obesity had no predictive role in the occurrence or time of BCR in the present study. Given the small number of patients in this subgroup, this exploratory analysis needs validation in prospective studies.

One of the main endpoints was to analyze the response to ADT in PwO. Obesity has been hypothesized to induce worse outcomes, either through insufficient suppression of testosterone levels due to altered drug tissue distribution or through facilitating the selection and development of cellular lines that thrive in a low testosterone environment. Castration resistance was seen in nearly half of our patients on ADT with sufficient follow-up data, after a median time of 14.5 months. There was no difference in the prevalence of obesity between the CRPC and CSPC groups, nor did obesity have a predictive role in the appearance of CRPC. However, in the group that developed resistance to ADT, obesity was associated with shorter time to castration resistance. To the best of our knowledge, we are the first to show this and the first to study the impact of obesity on ADT in a Romanian population. While the metabolic and cardiovascular adverse effects of ADT are well documented [9,10,11,12], there is little information on the effect of obesity on the response to ADT in PCa. Although it did not reach statistical significance, obesity was associated with a 3.8 times higher risk of developing CRPC in a retrospective study on 287 subjects conducted by Keto et al. [42], while the presence of metabolic syndrome was associated with shorter time to CRPC in another study [43]. The mechanisms that lead to castration resistance are complex and involve the androgen receptor (amplification that promotes hypersensitivity to low levels of testosterone; mutations that lead to activation by other molecules, such as progesterone, hydrocortisone, estradiol; overexpression of coactivators; ligand-independent androgen receptor activation through increased signaling; splice variants) or altered steroidogenesis due to adrenal production of dehydroepiandrosterone (DHEA) and DHEA-S which are converted to dihydrotestosterone through the alternative 5α-dione pathway, that bypasses testosterone and thus provide higher intra-tumoral androgen levels even in the context of ADT. Additionally, CRPC cell lines exhibit increased expression of HSD3B1, HSD3B2, HSD17B3, AKR1C3, and SRD5A1, steroidogenic enzymes that allow local production of androgens and steroids [44]. Whether or not obesity plays a role in one of these mechanisms remains to be determined. At the moment, studies on human prostatic cells and prostate tissue from mice with obesity induced by high fat diet have shown that overweight and obesity promotes and androgenic to estrogenic switch in the prostate by overexpression of estrogen receptor α, which is widely accepted to mediate the negative effects of estrogens (inflammation, proliferation and survival of cancer cells), and by inducing SRD5A2 promoter hypermetilation and inhibiting SRD5A2 expression, resulting in lower 5-α reductase activity [45,46].

The strengths of our study include the decently sized study population and the long-term follow-up data. Naturally, this study has several limitations; the retrospective nature lead to incomplete data regarding personal history of hypertension, diabetes, dyslipidemia; visceral adiposity was not assessed, neither by waist circumference measurement, nor by body composition analysis; information about the clinical T stage was missing in some patient’s electronic chart; serum testosterone measurements were not available for all patients on ADT; inflammation markers data were not available. These shortcomings will be addressed in a future prospective study we are planning to conduct in order to further validate our findings.

## 5. Conclusions

As obesity is reaching epidemic proportions worldwide, its role in cancer pathogenesis and evolution will become an important topic for future research. We have shown in the present study that weight and BMI are accessible tools that could be useful in determining the presence of metastatic disease and that obesity may be associated with shorter time to castration resistance in PCa patients with ADT. These findings illustrate the central part that lifestyle interventions should play in the management of such patients. PwO could benefit from a personalized therapeutic approach, and further studies are needed for a better understanding of the implications of obesity in PCa evolution, in order to provide adequate care to this growing category of patients.

## Figures and Tables

**Table 1:** Clinical and paraclinical characteristics of our study population.

Age, mean ± SD (years)	67.11 ± 8.05
Weight, median (IQR) (kg)	84.93 (20)
BMI, median (IQR) (kg/m^2^)	27.83 (6.02)
Obesity, N (%)	71 (27.6%)
Nutritional status, N (%) Underweight Normal weight Overweight Obesity class I Obesity class II Obesity class III	1 (0.4%) 66 (25.7%) 119 (46.3%) 52 (20.2%) 17 (6.6%) 2 (0.8%)
Hypertension, N (%)	116 (45.1%)
Dyslipidemia, N (%)	35 (13.6%)
Diabetes mellitus, N (%)	45 (17.5%)
Family history for neoplasia, N (%)	38 (14.8%)
PSA, median (IQR) (ng/mL)	12.8 (29.4)
Gleason Score, median (IQR)	7 (1)
Grade Group, N (%) I II III IV V Missing	41 (16%) 53 (20.6%) 47 (18.3%) 40 (15.6%) 44 (17.1%) 32 (12.5%)
Metastases at diagnosis, N (%)	59 (23%)
10-year all-cause mortality, %	14.9%

Abbreviations: BMI, body mass index; IQR, interquartile range; PSA, prostate-specific antigen; SD, standard deviation.

**Table 2:** Univariate comparative analysis according to nutritional status.

Parameter	Patients with Obesity N = 71 (27.6%)	Patients without Obesity N = 186 (72.4%)	*p*
Age, mean ± SD (years)	65.25 ± 6.96	67.82 ± 8.34	**0.022**
PSA, median (IQR) (ng/mL)	10.13 (22.71)	14.75 (34.73)	**0.016**
Gleason Score, median (IQR)	7 (1)	7 (1)	0.236
Grade group, N (%)			0.446
I–III	37 (52.1%)	104 (55.9%)	
IV–V	26 (36.6%)	58 (31.2%)	
Missing	8 (11.3%)	24 (12.9%)	
Metastases at diagnosis, N (%)	12 (16.9)	47 (25.3)	0.154

Abbreviations: IQR, interquartile range; PSA, prostate-specific antigen; SD, standard deviation. *p* values that show statistical significance are in bold.

**Table 3:** Univariate comparative analysis according to M stage at diagnosis.

Parameter	M1 N = 59 (23%)	M0 N = 198 (77%)	*p*
Age, mean ± SD (years)	67.64 ± 7.98	66.94 ± 8.08	0.559
10-year all-cause mortality (%)	37.5%	8.3%	**<0.001**
PSA, median (IQR) (ng/mL)	52.09 (119)	11 (16.95)	**<0.001**
Gleason Score, median (IQR)	8 (2)	7 (1)	**0.002**
Grade group, N (%) I–III IV–V Missing	20 (33.9%) 29 (49.2%) 10 (16.9%)	121 (61.1%) 55 (27.8%) 22 (11.1%)	<0.001 *
Weight, median (IQR) (kg)	76 (16)	83.5 (21)	**0.001**
BMI, median (IQR) (kg/m^2^)	26.3 (5.54)	27.14 (5.98)	**0.033**
Obesity, N (%)	12 (20.3)	59 (29.8)	0.154

Abbreviations: BMI, body mass index; IQR, interquartile range; M0, no metastases at diagnosis; M1, metastases present at diagnosis; PSA, prostate-specific antigen; SD, standard deviation. *p* values that show statistical significance are in bold; *, group “I–III” and group “IV–V” show statistical significance.

**Table 4:** Univariate comparative analysis of patients who underwent RP based on biochemical response.

Parameter	Biochemical Recurrence N = 17 (51.5%)	Biochemical Remission N = 16 (48.5%)	*p*
Age at diagnosis, mean ± SD (years)	64.31 ± 6.51	62.31 ± 5.61	0.359
Deceased, N (%)	1 (5.9)	1 (6.2)	0.965
PSA at diagnosis, median (IQR) (ng/mL)	11 (10.83)	8.21 (5.36)	0.081
Gleason Score, median (IQR)	7 (2)	7 (1)	0.526
Grade group, N (%) I–III IV–V Missing	11 (64.7%) 6 (35.3%) -	11 (68.8%) 4 (25%) 1 (6.2%)	0.599
Weight at diagnosis, median (IQR) (kg)	84 (19)	80 (20)	0.465
BMI at diagnosis, median (IQR) (kg/m^2^)	28.39 (4.94)	25.22 (5.92)	0.127
Obesity, N (%)	7 (41.2)	3 (18.8)	0.161
Time to recurrence, median (IQR) (months)	27.5 (48)	-	-

Abbreviations: BMI, body mass index; IQR, interquartile range; PSA, prostate-specific antigen; RP, radical prostatectomy; SD, standard deviation.

**Table 5:** Univariate comparative analysis of patients based on response to ADT.

Parameter	CRPC N = 38 (42.7%)	CSPC N = 51 (57.3%)	*p*
Age at diagnosis, mean ± SD (years)	66.45 ± 7	67.45 ± 6.83	0.499
10-year all-cause mortality rate (%)	37.1%	4.3%	**<0.001**
PSA at diagnosis, median (IQR) (ng/mL)	63.53 (132.8)	14.9 (26.4)	**0.001**
Gleason Score, median (IQR)	8 (2)	7 (1)	**0.006**
Grade group, N (%) I–III IV–V Missing	10 (26.3%) 21 (55.3%) 7 (18.4%)	33 (64.7%) 14 (27.5%) 4 (7.8%)	**0.001**
Weight at diagnosis, median (IQR) (kg)	80 (17)	84 (19)	0.243
BMI at diagnosis, median (IQR) (kg/m^2^)	26.64 (4.28)	27.31 (6.3)	0.709
Obesity, N (%)	7 (18.4)	18 (35.3)	0.080
Time to resistance, median (IQR) (months)	14.5 (14)	-	-

Abbreviations: ADT, androgen deprivation therapy; BMI, body mass index; CRPC, castration resistant prostate cancer; CSPC, castration sensitive prostate cancer; IQR, interquartile range; PSA, prostate-specific antigen; SD, standard deviation. *p* values that show statistical significance are in bold.

## Data Availability

The data presented in this study are available on request from the corresponding author.
